# Linking In Vitro Models of Endothelial Dysfunction with Cell Senescence

**DOI:** 10.3390/life11121323

**Published:** 2021-11-30

**Authors:** Francisco R. Jimenez Trinidad, Marta Arrieta Ruiz, Núria Solanes Batlló, Àngela Vea Badenes, Joaquim Bobi Gibert, Antoni Valera Cañellas, Mercè Roqué Moreno, Xavier Freixa Rofastes, Manel Sabaté Tenas, Ana Paula Dantas, Olga Tura-Ceide, Montserrat Rigol Muxart

**Affiliations:** 1 Cardiology Department, Institute Clinic Cardiovascular (ICCV), Hospital Clinic, Institute d’Investigacions Biomèdiques August Pi i Sunyer (IDIBAPS), University of Barcelona, 08036 Barcelona, Spain; frajimenez@clinic.cat (F.R.J.T.); marrieta@clinic.cat (M.A.R.); nsolanes@clinic.cat (N.S.B.); antonivalerac@gmail.com (A.V.C.); mroque@clinic.cat (M.R.M.); freixa@clinic.cat (X.F.R.); masabate@clinic.cat (M.S.T.); adantas@clinic.cat (A.P.D.); 2 Department of Pulmonary Medicine, Servei de Pneumologia, Hospital Clínic, Institut d’Investigacions Biomèdiques August Pi i Sunyer (IDIBAPS), University of Barcelona, 08036 Barcelona, Spain; avea@clinic.cat (À.V.B.); tura@clinic.cat (O.T.-C.); 3 Department of Cardiology, Erasmus MC, University Medical Center Rotterdam, 3015 Rotterdam, The Netherlands; j.bobiigibert@erasmusmc.nl; 4 Biomedical Research Networking Centre on Respiratory Diseases (CIBERES), CB06/06/0011 Group, Pulmonary Hypertension Programme, Instituto de Salud Carlos III, 28029 Madrid, Spain; 5 Department of Pulmonary Medicine, Santa Caterina Hospital de Salt and the Girona, Biomedical Research Institute (IDIBGI), Dr. Josep Trueta University Hospital de Girona, 17190 Girona, Spain; 6 Biomedical Research Networking Centre on Cardiovascular Diseases (CIBERCV), CB16/11/00358 Group, Arterial pathology, myocardial ischaemia and structural heart pathology Programme, Instituto de Salud Carlos III, 28029 Madrid, Spain

**Keywords:** senescence, endothelial dysfunction, starvation, oxidative stress, in vitro models, cell culture

## Abstract

Endothelial cell dysfunction is the principal cause of several cardiovascular diseases that are increasing in prevalence, healthcare costs, and mortality. Developing a standardized, representative in vitro model of endothelial cell dysfunction is fundamental to a greater understanding of the pathophysiology, and to aiding the development of novel pharmacological therapies. We subjected human umbilical vein endothelial cells (HUVECs) to different periods of nutrient deprivation or increasing doses of H_2_O_2_ to represent starvation or elevated oxidative stress, respectively, to investigate changes in cellular function. Both in vitro cellular models of endothelial cell dysfunction-associated senescence developed in this study, starvation and oxidative stress, were validated by markers of cellular senescence (increase in β-galactosidase activity, and changes in senescence gene markers SIRT1 and P21) and endothelial dysfunction as denoted by reductions in angiogenic and migratory capabilities. HUVECs showed a significant H_2_O_2_ concentration-dependent reduction in cell viability (*p* < 0.0001), and a significant increase in oxidative stress (*p* < 0.0001). Furthermore, HUVECs subjected to 96 h of starvation, or exposed to concentrations of H_2_O_2_ of 400 to 1000 μM resulted in impaired angiogenic and migratory potentials. These models will enable improved physiological studies of endothelial cell dysfunction, and the rapid testing of cellular efficacy and toxicity of future novel therapeutic compounds.

## 1. Introduction

The endothelium is the innermost cellular lining of all blood vessels, and an important initial barrier between the circulating blood, and its contents, and the extravascular compartment. Its main function is to maintain vascular homeostasis, including the regulation of coagulation, vascular tone, transport of nutrients, and metabolic by-products to and from the extravascular compartment, and physiological angiogenesis [1].

Endothelial dysfunction is linked with the risk factors associated with atherosclerosis, such as hypertension, diabetes, and dyslipidemia, among others [2]. The development of atherosclerotic plaques can result in highly disabling and fatal diseases, such as coronary artery disease, pulmonary thromboembolism, hypertension, peripheral arterial disease, chronic kidney failure, and stroke [3]. In addition to the impact on the individual person affected, endothelial dysfunction and its related pathologies have a significant socio-economic impact due to the high disability rate after cerebral and cardiovascular events triggered by this disease [4]. The generation of novel, representative in vitro models of this cellular dysfunction are required to better understand its pathophysiology, and to evaluate newly developed therapeutic targets for efficacy and cellular toxicity prior to evaluation using representative in vivo models.

Two main factors concomitant with the impairment of endothelial cells in vivo have been identified: (1) patients with endothelial cell dysfunction have an altered biochemical blood profile with excessive accumulation of cellular waste substances [5,6]; (2) there is increased production of reactive oxygen species (ROS), resulting in increased oxidative cellular stress [7]. Both these factors have been associated with cellular senescence [8,9].

Cellular senescence is a natural process characterized by a lack of cellular proliferation, and this can be exacerbated under conditions such as oxidative stress, inflammation, or abnormal cell growth, among others. Senescent cells, although not dividing, can still secrete factors which could be important in the development of deleterious cellular metabolic alterations [10,11]. Changes in the levels of P21 and sirtuins, or pro- and antisenescence proteins have been described as two specific molecular pathways associated with the impairment of endothelial cell functional capacities [11,12]. Senescent endothelial cells have been found in atherosclerotic lesions from patients with coronary artery disease, and could be a main driver of in vivo endothelial dysfunction [13,14,15]. Reproducing cellular senescence in vitro would enable more detailed evaluation of cell functions and molecular parameters occurring during this process.

Several studies have tried previously to develop in vitro models of endothelial cell dysfunction by exposing cells to changes in glucose concentration, increasing reactive oxygen species (ROS) or exposure to a range of pharmacological preparations known to adversely affect these cells [16,17,18]. However, although some studies have provided reproducible results relating endothelial cell dysfunction with cellular senescence [19,20], most have reported contradictory results [21,22,23].

The aim of this study was to subject endothelial cells in culture to common physiological stressors, such as nutrient restriction and oxidative stress, to standardize two in vitro models of endothelial cell dysfunction-associated senescence to provide a valid cell model to test the effects of putative endothelial protective compounds.

## 2. Materials and Methods

### 2.1. Cell Culture

Commercial Human Umbilical Vein Endothelial Cells (HUVECs) (Ref. P10961 Innoprot, Bizkaia, Spain; Ref. C0155C ThermoFisher Scientific, Waltham, MA, USA) were cultured under standard conditions (37 °C, 5% CO_2_, 21% O_2_, and 100% humidity) using DMEM:F12 (Ref. L0093 BioWest, Nuaille, France) supplemented with an LSGS Kit (Ref. S003K ThermoFisher Scientific, Waltham, MA, USA), 1% Penicillin/Streptomycin (Ref. 15140122 ThermoFisher Scientific, Waltham, MA, USA), and 10% fetal bovine serum (FBS) (Ref. 10500064 ThermoFisher Scientific, Waltham, MA, USA). The cell culture plates used were pre-coated with 0.2% gelatin (Ref. G1393-100ML Sigma-Aldrich, St. Louis, MO, USA) diluted in cell culture water (Ref. L0970-500 BioWest, Nuaille, France). Cells at passage 5 to 7, once they had achieved >90% confluence, were used for induction of cellular senescence after seeding into 96-well plates at a density of 1 × 10^4^ cells/well, unless described otherwise.

### 2.2. Induction of Cellular Senescence

HUVECs senescence was induced by two methods: (1) starvation or (2) increased oxidative stress. To induce starvation-associated senescence, cells were cultured at 37 °C, 5% CO_2_ for a total of 96 h, changing the medium at either 48, 72, or 96 h to represent different degrees of starvation compared to non-starved cells (negative control) for which the medium was changed every 24 h. For oxidative stress-associated senescence, cells were treated for 1 h with different concentrations of hydrogen peroxide (H_2_O_2_, at 200, 400, 600, 800, and 1000 μM) (Ref. 121076.1211 PanReac Applichem, Barcelona, Spain) diluted in DMEM w/o phenol red (Ref. 12-917F, Lonza, Basilea, Suiza) at standard conditions in a cell incubator. Cells not subjected to H_2_O_2_ were used as the negative control. After treatment, cells were incubated for 24 h in fresh standard medium prior to performing the assays, except for the determination of oxidative stress levels where the assay was performed immediately after the H_2_O_2_ treatment for 1 h, as ROS are rapidly quenched.

### 2.3. Cell Viability

HUVECs viability was determined with the Alamar Blue Assay (Ref. A50100 ThermoFisher Scientific, Waltham, MA, USA) according to the manufacturer’s instructions. Briefly, cells were incubated in commercial Alamar Blue reagent diluted (1:10) in supplemented DMEM with 5% FBS w/o phenol red for 2 h at 37 °C in darkness. Fluorescence intensity was read at 560 nm with a Synergy HTX Multi-Mode microplate reader (BioTek Instruments, Inc., Winooski, VT, USA). Data were reported as fold change of fluorescence intensity relative to the negative control cell preparation. Additionally, cell nuclei were labelled with DAPI (see Cell Senescence below) to count cell number per field, as another measure of cell viability.

### 2.4. Oxidative Stress Levels

To determine if H_2_O_2_ treatment was effective in producing oxidative stress on treated HUVECs, ROS production was assessed using specific dyes. Intracellular hydrogen peroxide (H_2_O_2_) and superoxide radical (O_2_^−^) were determined using 2,7-Dichlorofluorescein Diacetate (DCFHDA) and Dihydroethidium (DHE), respectively. Immediately after H_2_O_2_ treatment, cells were washed with PBS, and incubated with DCFHDA (50 μM) (Ref. D6883-50MG Sigma-Aldrich, St. Louis, MO, USA), DHE (50 μM) (Ref. D1168 ThermoFisher Scientific, Waltham, MA, USA), and Hoechst (30 nM) (Ref. H3570 ThermoFisher Scientific, Waltham, MA, USA) diluted in fresh supplemented DMEM w/o phenol red for 60 min. All procedures were performed in darkness. Fluorescence was read with a Synergy HTX Multi-Mode microplate reader (BioTek Instruments, Inc.,Winooski, VT, USA) at the following excitation/emission wavelengths: 518/606 nm for DHE; 495/520 nm for DCFHDA; and 350/461 nm for Hoechst. Microscopic visualization of fluorescent dyes was used as an internal control to verify the coherence of the data obtained with the spectrophotometer. DCFHDA and DHE fluorescence were normalized by Hoechst fluorescence, and data were reported as fold change of fluorescence relative to non-H_2_O_2_ treated cells.

### 2.5. Cell Senescence

β-Galactosidase activity, as a recognized marker of senescence [24], was evaluated using the β-Galactosidase Staining Kit (Ref. CS0030 Sigma-Aldrich, St. Louis, MO, USA) according to the manufacturer’s instructions. Briefly, HUVECs, 5 × 10^4^/well, were seeded into 12-well plates. After induction of senescence, cells were washed twice with phosphate buffered saline (PBS), fixed in 4% paraformaldehyde, and incubated overnight at 37 °C with an X-Galactosidase solution in a rocking incubator. To quantify cell number per field, cells were then washed twice with PBS for 5 min, and stained with 3 nM DAPI (Ref. D9542-10MG Sigma-Aldrich, St. Louis, MO, USA) for 15 min at room temperature. Two wells for each different condition were analyzed in every plate, and five pictures of representative areas per well were taken at 100×. Blue-stained nuclei were considered positive for a cell. Images were analyzed using ImageJ (NIH, Bethesda, MD, USA), and results reported as the derived mean percentage of positive β-Galactosidase cells per field.

### 2.6. RNA Isolation, Retro Transcription, and Quantitative PCR

HUVECs were seeded into 6-well plates at a density of 1 × 10^5^ cells/well. After induction of senescence, cells were removed by trypsin solution, centrifuged (500× *g*, 5 min), and the resultant dry pellets stored at −80 °C until the extraction of RNA was performed. Total RNA was extracted using Qiazol (Ref. 79306, Qiagen, Hilden, Alemania) and the miRNeasy Mini Kit (Ref. 217004, Qiagen, Hilden, Alemania). Subsequently, 500 ng of RNA from cell lysates was retrotranscribed to cDNA using a High-Capacity cDNA Reverse Transcription Kit (Ref. 4368814, ThermoFisher Scientific, Waltham, MA, USA) and the following program: 10 min at 25 °C; 10 min at 37 °C; and 5 min at 85 °C.

To assess the levels of expression of key senescence cell markers, anti-senescence sirtuin 1 (SIRT1), pro-senescence cyclin-dependent kinase inhibitor 1 (P21), and tumor protein p53 (TP53) genes were analyzed using quantitative PCR (qPCR) by a ViiA 7 Real-Time PCR System (ThermoFisher Scientific, Waltham, MA, USA) with PowerTrack™ SYBR™ Green Master Mix (Ref. A46109, ThermoFisher Scientific, Waltham, MA, USA). Beta-Actin (ACTB) was used as a housekeeping gene. All primers were purchased from ThermoFisher Scientific, Waltham, MA, USA. Details of primer sequences are listed in Table 1.

### 2.7. Migration Capacity

To study the migratory capacity of HUVECs after induction of senescence, a wound healing assay was performed on cells at confluence in a 24-well plate. Briefly, one scratch across the endothelial cells for every condition and replicate was made using a 200-microliter pipette tip. Pictures from the same zone of the scratch were taken at 50× magnification using a Zeiss Axiovert microscope (Carl Zaiss, Oberkochen, Germany) at 0, 6, 12, and 24 h after the insult. Wound areas were analyzed using ImageJ (NIH, Bethesda, MD, USA), and data was reported as the percentage of wound closed at a determined time calculated according to the following Formula (1):

(1) $$\%\;\mathrm{Closed}\;\mathrm{Wound}\;\mathrm{Area}\;\left(t=x\right)=\frac{\mathrm{Wound}\;\mathrm{Area}\;\left(t_{0}\right)-\mathrm{Wound}\;\mathrm{Area}\;\left(t_{x}\right)}{\mathrm{Wound}\;\mathrm{Area}\;\left(t_{0}\right)}\times 100$$

### 2.8. Angiogenesis Capacity

To study the vascular network formation capacity of HUVECs after starvation and exposure to H_2_O_2_, a 2D angiogenesis assay was performed. Cells were trypsinized and seeded onto matrigel-coated (Ref. A1413202 ThermoFisher Scientific, Waltham, MA, USA) angiogenesis μ-slides (Ref. 81506 Ibidi, Gewerbehof Gräfelfing, Germany) at a density of 2 × 10^4^ cells/well according to the manufacturer’s instructions. Pictures (at 50× magnification) from every well were taken with a Zeiss Axiovert microscope 12 h after seeding, and analyzed using ImageJ. Data were presented as mesh number, mesh area, segment number, segment length, number or branching points, number of ramifications, and percentage of extremes per branching point.

### 2.9. Statistical Analysis

Results are reported as mean ± standard deviation. Data normality was assessed with the Shapiro–Wilk Test, and the differences among means were analyzed by one-way ANOVA with Dunnett’s post hoc for multiple comparisons. Pearson’s correlation test and linear regression was used to analyze the correlation between H_2_O_2_ concentration and viability, and DCFHDA and DHE fluorescence. Data were analyzed and plotted using GraphPad Prism v9 (GraphPad Software Inc., La Jolla, CA, USA). Significant differences were considered when *p*-value ≤ 0.05.

## 3. Results

### 3.1. Cell Viability

**Figure 1:**
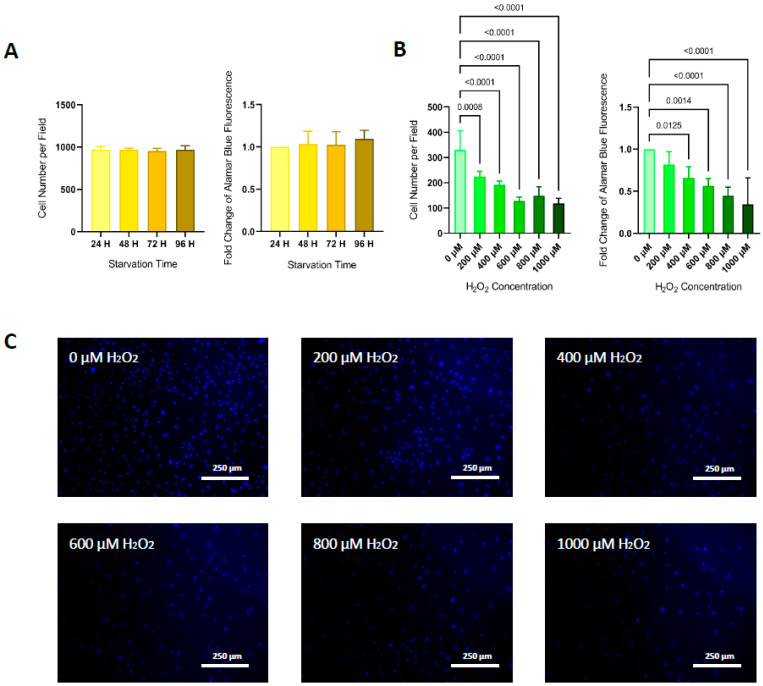
Cell viability. (**A**) HUVECs subjected to different times of starvation showed no changes in cell number per field (*p-ANOVA* = 0.8976), nor Alamar blue fluorescence intensity (*p-ANOVA* = 0.6339) compared to negative control HUVECs. (**B**) HUVECs subjected to increasing doses of H_2_O_2_ showed a significant reduction in cell viability, compared to non-treated cells, both in cell number per field (*p-ANOVA* < 0.0001) and Alamar blue fluorescence intensity (*p-ANOVA* < 0.0001). Bar charts represent mean ± SD of five independent experiments. Differences in means were compared by ANOVA followed by Dunnett’s post hoc test. Significance was considered when *p* < 0.05. (**C**) Staining of cell nuclei with DAPI (blue fluorescence) showed a progressive reduction in cell number per field as H_2_O_2_ concentrations increased.

Endothelial cells subjected to different durations of starvation did not show any differences in viability when assessed by cell number per field nor Alamar blue fluorescence intensity (Figure 1A)**.** Cells treated with different concentrations of H_2_O_2_ showed a significant reduction in cell viability compared to non-treated cells, both in cell number per field for all H_2_O_2_ doses, and Alamar blue fluorescence intensity for concentrations ≥ 400 μM (Figure 1B,C). There was a significant inverse correlation between the amount of H_2_O_2_ and cell viability (*p* < 0.05 for cell number per field H_2_O_2_ concentration correlation, and *p* < 0.001 for Alamar blue fluorescence intensity H_2_O_2_ concentration correlation).

### 3.2. ROS Generation

**Figure 2:**
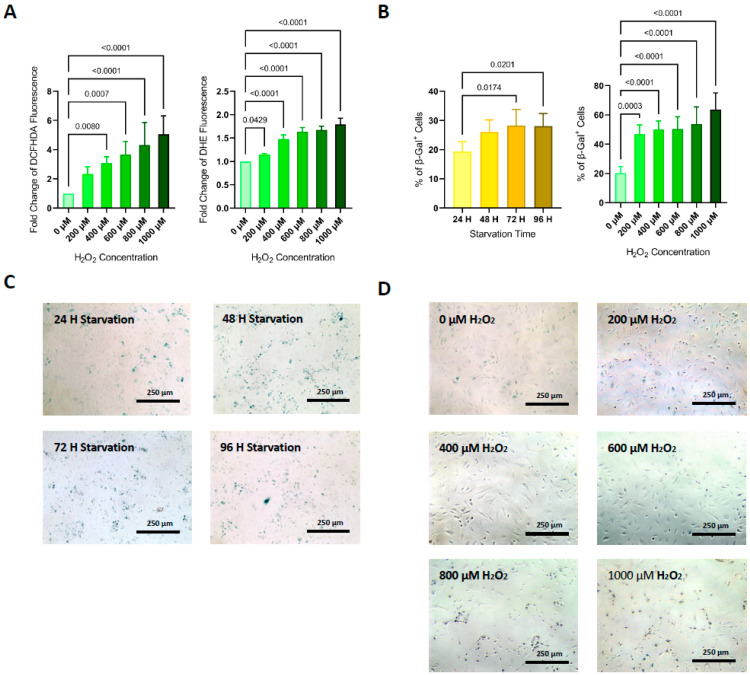
ROS production and β-Galactosidase activity. (**A**) Note progressive significant increases in DCFHDA and DHE (*p-ANOVA* < 0.0001, both assays) fold change after subjecting HUVECs to different doses of H_2_O_2_ compared with HUVECs not subjected to H_2_O_2_ (negative control). (**B**) Note increases in cellular senescence in HUVECs, measured as a percentage of β-galactosidase-stained cells per field, after increasing times of starvation and exposure to different concentrations of H_2_O_2_ (*p-ANOVA* < 0.0001 and = 0.0214, respectively). Bar charts represent mean ± SD of 5 independent experiments. Differences in means were compared by ANOVA followed by Dunnett’s post hoc test. Significance was considered when *p* < 0.05. Representative images showing an increase in the number of blue-stained cells (β-Galactosidase positive) after augmenting starvation times (**C**) and H_2_O_2_ concentrations (**D**).

The intracellular levels of ROS (determined by DCFHDA fluorescence) measured in HUVECs treated with H_2_O_2_ were significantly higher for concentrations ≥ 400 μM compared with non-treated cells. O_2_^−^ radical (determined by DHE fluorescence) measured in the same cells increased significantly for all H_2_O_2_ concentrations (Figure 2A). A significant positive correlation with the concentration of the H_2_O_2_ used was found for both ROS and O_2_^−^ levels (*p* < 0.001 and *p* < 0.05, respectively).

### 3.3. Cell Senescence

Compared with non-starved HUVECs, β-Galactosidase staining of cells after 72 and 96 h of starvation showed a higher percentage of positive cells (19. 4 ± 4.2% vs. 28.2% ± 5.6% and 28.0% ± 4.4%, respectively; *p* < 0.05) (Figure 2B,C). HUVECs treated with every chosen H_2_O_2_ concentration showed a significant increase of β-Galactosidase positive cells when compared to non-treated HUVECs (Figure 2B,D).

### 3.4. Transcriptional Changes

**Figure 3:**
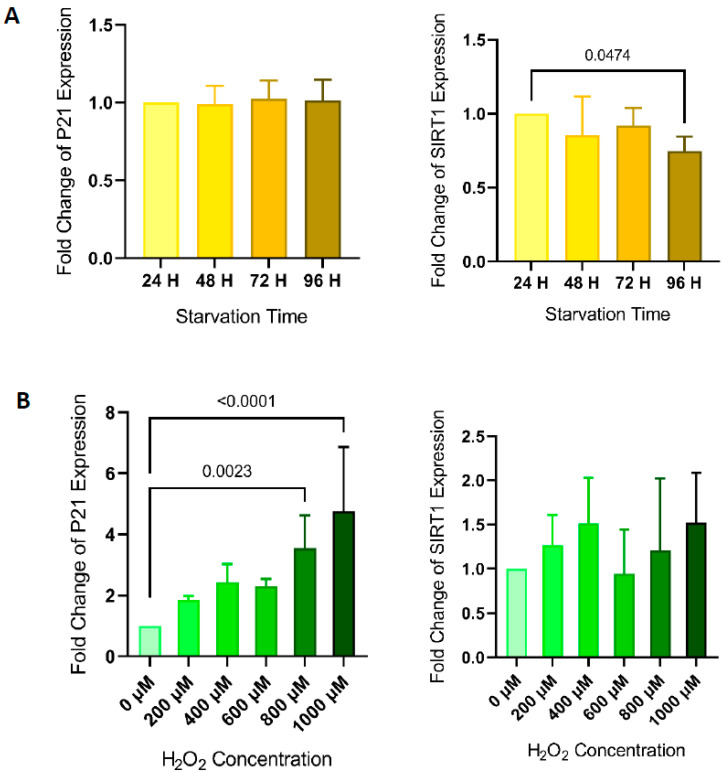
Levels of expression of different markers of senescence in HUVECs. Bar charts show changes in transcription levels of expression of SIRT1 and P21 in HUVECs assessed by quantitative PCR after being subjected to different times of starvation (*p-ANOVA* = 0.1015 and *p* = 0.1919, respectively) (**A**) or after being subjected to increasing concentrations of H_2_O_2_ (*p-ANOVA* = 0.3811 and *p* < 0.0001, respectively) (**B**). Bar charts represent mean ± SD of 5 independent experiments. Differences in means were compared by ANOVA followed by Dunnett’s post hoc test. Significance was considered when *p* < 0.05.

No significant changes in the expression of P21-specific mRNA were detected in HUVECs subjected to starvation. After HUVECs were subjected to 96 h of starvation, a significant reduction of the expression of SIRT1-specific mRNA was detected (0.75-fold, *p* < 0.05) (Figure 3A). H_2_O_2_ treatment of HUVECs at concentrations of 800 and 1000 μM significantly increased the expression of P21-specific mRNA (3.5-fold, *p* < 0.05; and 4.5-fold, *p* < 0.001, respectively), but no changes in SIRT1 mRNA expression were detected in response to exposure to H_2_O_2_ (Figure 3B).

Under our experimental conditions, levels of TP53-specific mRNA were almost undetectable (ct ≥ 35) in most samples from all groups (data not shown), making any comparisons invalid.

### 3.5. Functional Capacities: Migration and Angiogenesis

**Figure 4:**
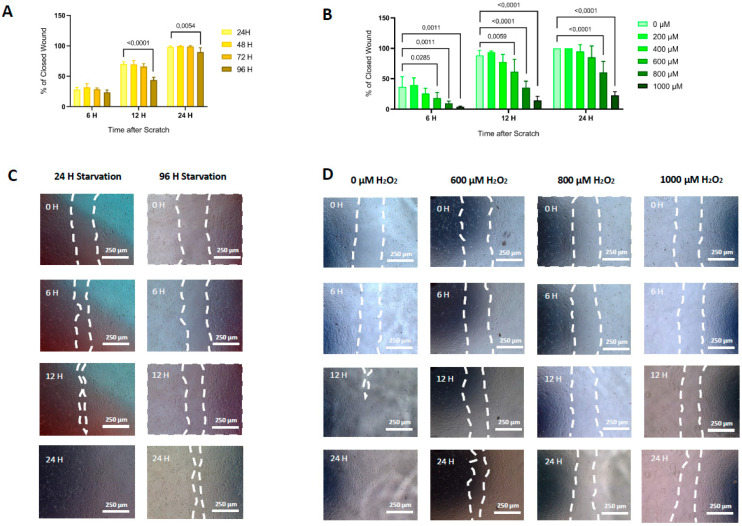
Migration capacity. Time-course of the percentage of closed wound area after scratch in cultured HUVECs submitted to different periods of starvation (*p*-ANOVA = 0.0659 at 6 h after scratch, *p* < 0.0001 at 12 h, and *p* = 0.0018 at 24 h) (**A**) or treatment with increasing concentrations of H_2_O_2_ (*p-ANOVA* < 0.0001 at 6 h, *p* = 0.4181 at 12 h, and *p* = 0.1026 at 24 h) **(B).** Bar charts represent mean ± SD of 5 independent experiments. Differences in means compared by ANOVA followed by Dunnett´s post hoc test. Significance was considered when *p* < 0.05. Representative images of wound healing assay illustrate a delay in wound area closure over time in cells with 96h of starvation (**C**) and those subjected to different concentrations of H_2_O_2_ (**D**) compared to untreated cells (negative control), respectively. White dotted line indicates the wound border after the scratch.

Compared with non-starved HUVECs, cells subjected to 96h of starvation showed a significant decrease in the area of wound closure at 12 h (70.1% ± 3.7% vs. 65.8% ± 4.6%, respectively; *p*-value < 0.0001) and at 24 h (98.4% ± 1.7% vs. 89.6% ± 7.2, respectively; *p*-value = 0.0054) after wound induction (Figure 4A,C). Following oxidative stress treatment, a significant decrease of the percentage of area of closed wound relative to non-treated cells was observed at 6 and 12 h after the start of the assay for concentrations of H_2_O_2_ ≥ 600 μM, and at 24 h for concentrations ≥ 800 μM (Figure 4B,D).

**Figure 5:**
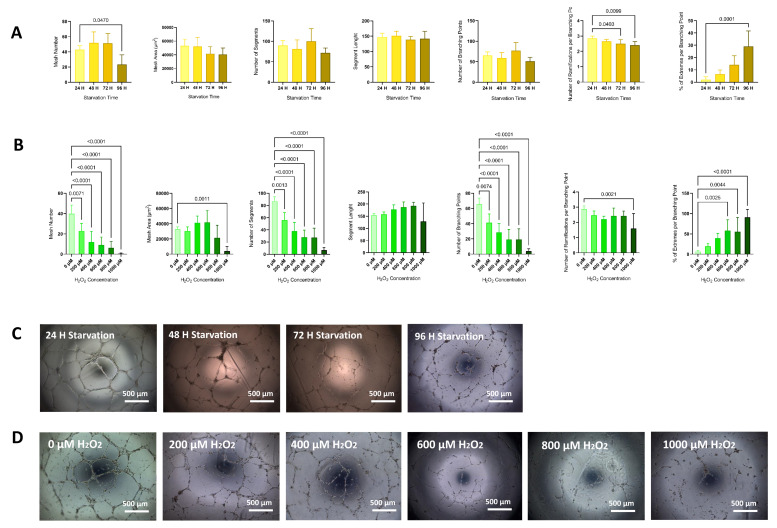
Angiogenesis. Differences in mesh number, mesh area, segment number, segment length, number of branching points, and number of ramifications and percentage of extremes per branching point analyzed after 2D angiogenesis assay on Matrigel with HUVECs (*p-ANOVA* < 0.0001, *p* < 0.0001, *p* < 0.0001, *p* = 0.0566, *p* < 0.0001, *p* = 0.0150, and *p* < 0.0001, respectively) subjected to different periods of starvation (**A**), or subjected to increasing concentrations of H_2_O_2_ (*p-ANOVA* = 0.0049, *p* = 0.1535, *p* = 0.2194, *p* = 0.6096, *p* = 0.0512, *p* = 0.0191, and *p* = 0.0002, respectively) (**B**). Bar charts represent mean ± SD of 5 independent experiments. Differences in means was determined by ANOVA followed by Dunnett´s post hoc test. Significance was considered when *p* < 0.05. Representative images show a reduction of angiogenesis capacity after different periods of starvation (**C**) or subjected to increasing concentrations of H_2_O_2_ (**D**).

Compared with non-starved HUVECs, those subjected to 96 h of starvation had a reduction in angiogenesis as denoted by a decrease in the mesh number (42.9 ± 5 vs. 23.3 ± 12.6, respectively; *p* < 0.05), the number of ramifications per branching point (2.9 ± 0.15 vs. 2.4 ± 0.23, respectively; *p* < 0.05), and an increase in extremes per branching point (2 ± 2.345 vs. 29 ± 12.75, respectively; *p* < 0.001). When compared with non-starved HUVECs, cells subjected to 72h of starvation also showed a significant reduction of number of ramifications per branching point (2.9 ± 0.15 vs. 2.5 ± 0.3, respectively; *p* < 0.05). No significant changes in mesh area, segment number, and length or number of branching points were detected between any timepoints (Figure 5A,C).

HUVECs treated with H_2_O_2_ showed a significant reduction in mesh number, number of segments, and number of branching points for all H_2_O_2_ concentrations, as well as an increase in the percentage of extremes per node for concentrations of H_2_O_2_ ≥ 600 μM when compared with HUVECs not treated with H_2_O_2_. Cells treated with 1000 μM of H_2_O_2_ showed a reduction of mesh area and number of ramifications per branching point when compared with non-treated cells. No significant changes in segment length were found for any H_2_O_2_ concentration (Figure 5B,D).

## 4. Discussion

This study describes two in vitro models of endothelial cell dysfunction both validated by assessing endothelial cell viability, ROS generation, migration, and angiogenesis abilities. The models showed significant changes in many of the parameters examined, including the transcriptional analysis which demonstrated the relationship between endothelial cell dysfunction, and altered cell viability with increased senescence, as well as impaired migratory and angiogenic properties. Both models are inexpensive, rapid, reliable, easy to perform, and validated on parameters indicative of the main recognized drivers and/or outcomes of endothelial cell dysfunction.

To date, different endothelial cell dysfunction-associated senescence models have been described using a variety of cytokines, pharmaceutical compounds, metabolic by-products, or cellular stress induced by other methods. However, most studies were not standardized, and based on very specific pathways that do not represent the complexity of the environment in which endothelial dysfunction occurs. One of the most described methods to study the functional and mechanistic alterations of cellular senescence is based on replicative senescence, that is, simulating senescence while measuring perturbations during repetitive cell division. Nonetheless, this method has reported highly variable results, and required very long culture times to induce senescence [19,20,23]. The latter potentially compromising the model due to the possibility of confounding complications associated with prolonged in vitro culture.

Using HUVECs, we determined the optimum time to induce senescence and endothelial cell dysfunction by simulating starvation was 96 h. At this time point, cell viability was unaffected, but cells showed significant changes in recognized markers of senescence, including decreased angiogenic and migratory capabilities. This model of cell starvation, based on gradual restriction of culture media replacement, resulting in progressive nutrient constraint and accumulation of waste substances, seeks to be more physiologically representative than other current endothelial cell starvation models described, which are mainly based on a single reduction of serum percentage in cell culture media. For example, Jeong et al. showed how glutamine deprivation and/or reduction of serum percentage induced a stress response in HUVECs denoted by increasing levels of active caspases, and a resultant decrease in cell viability as measured by metabolic parameters. However, their model did not evaluate functional capacities or senescence of HUVECs [25]. In contrast, in our model, in which cell viability was unaffected, we found that migratory and angiogenic properties of deprived HUVECs, as well as SIRT1 (an anti-aging protein) expression decreased after 96 h of starvation, and β-galactosidase activity, a marker of senescence, increased after 72 h and further by 96 h. Our results, in agreement with a previous study by Potente et al. [13], suggest that the reduction in SIRT1 levels could be the main driver reducing the functional capacities of endothelial cells through modification of SIRT1-mediated transcription factors activities. Other studies have reported changes in functional or structural capacities when HUVECs were cultured under a reduced percentage of serum: Chung Lim et al. have seen a reduction of primary cilia, a blood biochemical and mechanoreceptor of endothelial cells [26]; Yi-Xin et al. observed an increase in the number of cells in GO/G1 phases of cell division [27]; and Lake et al. demonstrated a reduction in angiogenic capacity [28]. However, a reduction in the percentage of serum is not a physiologically representative way to simulate stress because this situation is rare in vivo. In addition, the small number of studies investigating starvation and endothelial dysfunction, and that simulation of starvation has usually been used to synchronize cells or to avoid the effect of FBS on the activation of certain molecular pathways before treatments, suggests serum reduction is a physiologically unrepresentative method of induction of endothelial dysfunction in vitro. Furthermore, the lack of a standardized methodology hinders the comparison of data from different research groups. However, the results obtained in the present study provide a novel, affordable, and consistent, physiologically representative method to induce starvation-associated senescence and endothelial dysfunction that could be easily reproduced by different laboratories.

Inducing dysfunction-related senescence by exposure of HUVECs in vitro to H_2_O_2_ simulates the oxidative stress suffered by endothelial cells in people with endothelial cell dysfunction. The results showed a significant reduction of cell viability, migration, and angiogenic capacity, as well as a significant increase in senescence markers and ROS species in a concentration-dependent manner when H_2_O_2_ were used. Previous studies have used H_2_O_2_ to induce senescence in HUVECs: Song et al. used a concentration of 60 μM H_2_O_2_, and observed changes in senescence markers (such as increased β-Galactosidase activity, and reduction of SIRT1 expression) and anabolic pathways after treatment [22]. Unfortunately, no cell viability or functional assays were reported, and, thus, no relation between senescence and cell dysfunction could be inferred. Suo et al. treated HUVECs with 25 μM H_2_O_2_, and analyzed cell viability and migration capacity, but no concentration curve for cell viability and senescence assays was provided [23], and no other studies support their results at this concentration of H_2_O_2_. Several studies used H_2_O_2_ to induce cytotoxicity and dysfunction on HUVECs, describing IC50 over a range of concentrations, including 60 μM [22], 300 μM [29], higher than 300 μM [30], and of 800 μM [31]. These studies analyzed ROS concentrations, ROS-related enzyme levels, and molecular changes, but only Song et al. analyzed senescence markers, and at only one of the concentrations of H_2_O_2_ [22]. Unfortunately, none of these studies undertook functional assays. In the present study, we have assessed the function and viability of H_2_O_2_-treated HUVECs, describing a significant impairment of HUVECs migratory and angiogenic capacities, as well as reporting cell viability for every concentration of H_2_O_2_ to which HUVECs were exposed. Additionally, we observed a significant increase in β-Galactosidase activity, as well as a significant increase of P21 expression, but no differences in SIRT1 expression. The effects of H_2_O_2_ on HUVECs could be explained, partially, by the increase in expression of P21 because of its association with cell cycle arrest and induction of cell senescence [12]. Differences in results in the current literature despite using identical concentrations of H_2_O_2_, and similar results but at different concentrations of H_2_O_2_ may be due to differences in cell passage, medium used, the presence of confounding antioxidants, such as phenol red, or the concentration of FBS [32,33].

Although a large number of publications have used HUVECs exposed to H_2_O_2_ as a model of oxidative stress and senescence, most focused on investigating the role of treatments [17,22] to either prevent senescence or decrease oxidative stress. Only a few attempted to standardize an optimal dose of H_2_O_2_ [21,29,30,31], and none of these studies addressed endothelial dysfunction-related senescence only. Therefore, the H_2_O_2_-induced endothelial dysfunction-related senescence model presented in this study has shown the importance of H_2_O_2_ concentrations on HUVECs using a defined medium and protocol with respect to obtaining reproducible results. Moreover, all functional assays and senescence markers have been analyzed for all H_2_O_2_ concentrations studied in order to determine the optimum relationship between cytotoxicity, senescence, and dysfunction. Various other studies subjected endothelial cells to different compounds to induce stress, such as ethanol [34] or glucose [15,35], among others, without providing evidence that the concentration and duration of exposure were optimal. Therefore, some studies may have underestimated the effects of treatments because the cellular dysfunction observed was not significant, and, even if the results were not biased, validation of any model is required prior to its use investigating the efficacy of novel treatments.

The starvation model developed in the present study could be especially useful to test novel compounds to prevent or reduce cell senescence and endothelial dysfunction. This model has the advantage of not affecting cell viability, so large cell numbers of cells are not required, and the studies are not confounded by the effects of apoptosis. To test the recovery of cells after induction of senescence, future studies would simply refresh the medium at the chosen time point. The model of oxidative stress developed in this study does result in a concurrent reduction in cell viability and functional capacities, as well as increased concentrations of ROS and levels of senescence markers at concentrations of 600 to 1000 μM H_2_O_2_. This model could be useful for assessing compounds that may potentially reduce apoptosis, ROS generation, and senescence development, as well as their abilities to rescue cells undergoing oxidative stress-related changes. Both models result in changes in angiogenic parameters and transcriptional changes, suggesting that both types of stress produce their effects through the modification of specific molecular pathways. Further studies that identify the specific molecular pathways and changes in response to different types of stress and their roles on endothelial cell dysfunction would be of value.

## 5. Limitations and Future Studies

The effect of changing the medium after 96 h of starvation has not been studied because the objective of this research was to generate models of endothelial cell dysfunction-related senescence, not to evaluate the rescue of cells after induction of senescence. However, the recovery of the starved cells after changing the medium must be considered for studies that want to investigate the efficacy of potential compounds for this use. In contrast to other studies on cellular senescence, in our starvation-induced senescence model, no changes in p21 (a key marker of senescence) were observed. However, in our model, the results showed that senescence was associated with a decrease in sirtuins, suggesting different specific molecular pathways of action. As the results presented in this study were obtained using HUVECs, future studies adapting our models to other endothelial cell types, such as arterial or microvascular endothelial cells, may need modification, given the particular physiological characteristics of each endothelial cell type.

## 6. Conclusions

These validated, highly reliable, easy to establish and perform models undertaken with commercial human cells subjected to physiological stress will enable improved studies of the mechanisms of endothelial cell dysfunction, and allow direct comparison of results between different research groups. In addition, our models will enable more rapid, cost-effective assessment of the efficacy and cellular toxicity of future novel therapeutic compounds to treat the main pathologies related to endothelial dysfunction.

## Figures and Tables

**Table 1:** Primer Sequences for quantitative PCR.

Gene.	Forward Sequence 5′ to 3′	Reverse Sequence 5′ to 3′
SIRT1 (NM_012238.4)	5′-GAGGAGGCGAGGGAGGAG-3′	5′-GCTGAAGGGCGAGGGC-3′
P21 (NM_000389.4)	5′-AGTCAGTTCCTTGTGGAGCC-3′	5′-CATTAGCGCATCACAGTCGC-3′
TP53 (NM_000546.5)	5′-GGAGCCGCAGTCAGATCCTA-3′	5′-GGAATTTTCGCTTCCCACAGG-3′
ACTB (NM_001101.3)	5′-CCTCGCCTTTGCCGATCC-3′	5′-GCTCGATGGGGTACTTCAGG-3′

SIRT1 = sirtuin 1; P21 = cyclin-dependent kinase inhibitor 1; TP53 = tumor protein p53; and ACTB = Beta-Actin.
